# Control of alternative end joining by the chromatin remodeler p400 ATPase

**DOI:** 10.1093/nar/gkv1202

**Published:** 2015-11-17

**Authors:** Gemael-Cedrick Taty-Taty, Catherine Chailleux, Muriel Quaranta, Ayeong So, Josée Guirouilh-Barbat, Bernard S. Lopez, Pascale Bertrand, Didier Trouche, Yvan Canitrot

**Affiliations:** 1 Université de Toulouse, UPS, LBCMCP, F-31062 Toulouse, France; 2 CNRS UMR5088, LBCMCP, F-31062 Toulouse, France; 3 Université Paris Sud, CNRS UMR8200, IGR, Villejuif, France; 4 CEA DSV, UMR 967 CEA-INSERM-Université Paris Diderot-Université Paris Sud, Fontenay aux roses, France

## Abstract

Repair of DNA double-strand breaks occurs in a chromatin context that needs to be modified and remodeled to allow suitable access to the different DNA repair machineries. Of particular importance for the maintenance of genetic stability is the tight control of error-prone pathways, such as the alternative End Joining pathway. Here, we show that the chromatin remodeler p400 ATPase is a brake to the use of alternative End Joining. Using specific intracellular reporter susbstrates we observed that p400 depletion increases the frequency of alternative End Joining events, and generates large deletions following repair of double-strand breaks. This increase of alternative End Joining events is largely dependent on CtIP-mediated resection, indicating that it is probably related to the role of p400 in late steps of homologous recombination. Moreover, p400 depletion leads to the recruitment of poly(ADP) ribose polymerase (PARP) and DNA ligase 3 at DNA double-strand breaks, driving to selective killing by PARP inhibitors. All together these results show that p400 acts as a brake to prevent alternative End Joining-dependent genetic instability and underline its potential value as a clinical marker.

## INTRODUCTION

DNA double strand breaks (DSB) are DNA damages with different origins that need to be repaired to avoid cell death. In addition, their repair must be faithful to prevent genetic instability such as chromosome rearrangements. The repair of DSB is performed by two major pathways: homologous recombination (HR), which is cell cycle dependent as it uses the information present on sister chromatid, and non-homologous end-joining (NHEJ). In mammalian cells, NHEJ is the main pathway involved in DSB repair since it functions throughout all cell cycle phases (1,2). HR begins by the generation of single strand DNA through DNA resection mediated by the exonuclease activity of Mre11 but also CtIP, Exo1 or Dna2 (3). In S and G2 cells, the two pathways co-exist, and the extent of DNA resection is an important parameter dictating the use of one or the other pathway. When resection is important, DSBs can no longer be repaired by classical NHEJ and have to be repaired by HR. NHEJ involves direct sealing of the DNA ends made of the sequential events of Ku binding to DNA ends followed by DNA-PKcs recruitment and final ligation performed by the ligase IV–XRCC4 complex (4). However, when classical NHEJ is defective, DSB are repaired unfaithfully with the generation of large deletion at the site of repair (5). This repair activity was named alternative NHEJ (AltEJ). As an error-prone mechanism, AltEJ has been proposed to be involved in the chromosomal rearrangements observed in some leukemia (6), although some recent findings suggest that classical NHEJ could also be involved (7). Despite its importance for genetic instability, the factors involved in the AltEJ process are still under debate and the control of its activity is largely unknown.

More and more evidence indicate that DNA repair is facilitated or inhibited depending on the chromatin context (8,9). DNA is wrapped onto histone proteins to form nucleosomes and this structure can be modified by changing the composition of the nucleosome (introduction of histone variant) and/or by the post translational modifications of histones such as acetylation and methylation. Modulation of DNA repair activity has been shown to be associated or influenced by changes in chromatin marks (10) or by the activity of chromatin remodelers such as INO80 (11), CHD4 (12) or ACF1 (13). Recent data have shown the importance of the p400 ATPase, an enzyme conserved from yeast to human and which can mediate the incorporation of the histone variant H2A.Z in chromatin. p400 belongs to a multimolecular complex also containing the histone acetyl transferase Tip60. We showed that the p400 ATPase promotes HR by binding directly the Rad51 recombinase (14). Other studies indicate that it also affects NHEJ by controlling Ku recruitment to DSB via the incorporation of the histone variant H2A.Z (15). However, the role of H2A.Z in DSB repair in mammals is still a matter of debate, and as a consequence, the exact role of p400 is unclear (16,17). Here, we examined DSB repair activity in p400 depleted cells. We observed that p400 promotes genetic stability by preventing the use of Alt-EJ.

## MATERIALS AND METHODS

### Cell culture and transfections

The GC92 and GCSH14 cell lines have been derived from SV40 T-transformed GM639 human fibroblasts. The U2OS-EJ2 cell line has been derived from the osteosarcoma cell line U2OS (18). All cell lines were grown at 37°C in Dulbecco's modified Eagle's medium (DMEM) supplemented with antibiotics, 10% FCS (all from Invitrogen). The AsiSI-ER-U2OS stable cell line (19) was cultured in DMEM medium supplemented with10% FCS and antibiotics. When needed, 300 nM of 4OH-tamoxifen was added to culture medium for 4 h.

For siRNA transfection, 1 × 10^5^ cells were transfected with siRNA (10 nM) using Interferin (Ozyme, France) according to the manufacturer's instructions, or 5 × 10^6^ cells were electroporated with siRNAs (10 μM) using an electroporation device (Amaxa AG, Koln, Germany), according to manufacturer's specifications (for all other experiments). Plasmids were transfected with Jet-PEI (Ozyme, France) according to the manufacturer's indications.

### Western blot

Total cell lysates were prepared by the resuspension of the cells directly in Laemmli buffer and sonication. Cells extracts were separated on 4–12% gradient SDS-PAGE. Proteins were transferred on nitrocellulose membrane. Primary antibodies as well as peroxidase-conjugated secondary antibodies were used according to standard western blot procedure and peroxidase activity was detected by using the Lumi-LightPLUS Western Blotting Substrate (Roche Diagnostics, Meylan, France). The antibodies used were anti p400 from Abcam, α-tubulin from Sigma-Aldrich, anti myc from Roche (9E10).

### NHEJ assay

GCSH14 cells were transfected with siRNA (10 nM) using Interferin (Ozyme) according to the manufacturer's instructions. After 24 h, cells were transfected with I-SceI coding plasmid using JetPei (Ozyme) according to the manufacturer's instructions. After 72 h, cells were trypsinized and analyzed by flow cytometry (BD Facscalibur) to detect GFP expressing cells. Percentage of GFP positive cells was calculated after analysis on 25 000 sorted events. Measurement of NHEJ events using the GC92 cell line were performed as previously described (20,21). Cells were transfected with the different siRNA and I-SceI plasmid as described for GCSH14 cell line. The presence of CD4 events was detected using antibody coupled to alexa488 directed against CD4 (Biolegend). CD4 positive cells were quantified by flow cytometry on 25 000 sorted events.

### Junction sequence analysis

Genomic DNA from GC92 cells treated with the different siRNAs and transfected with I-SceI expression plasmid was prepared using DNA easy kit (Qiagen) according to the manufacturer's instructions. PCR were performed on the different genomic DNA using primers CMV1 (5′-TGGCCCGCCTGGCATTATGCC-3′) and CD4int (5′-GCTGCCCCAGAATCTTCCTCT-3′). PCR products were cloned into pGEM-T (Promega) and individual clones sequenced (Eurofins, Ebersberg, Germany).

### Measurement of resection at DSB

U2OS-ASiSI cells (DSB Inducible via AsiSI) (19) were transfected with siRNA using the Cell Line Nucleofactor kit V (Amaxa) according to the manufacturer's instructions. After 48 h, siRNA transfection, cells were treated or not with 300 nM of 4-hydroxytamoxifen (4OHT) (Sigma; H7904) for 4 h. DNA was purified using QIAGEN DNeasy kit. The level of resection generated at an AsiSI-induced DSB was measured by quantitative polymerase chain reaction (qPCR). After RNaseH treatment (20 units), genomic DNA sample was digested or not with 20 units of restriction enzymes (BanI) at 37°C overnight. Two microliters of digested or not samples (20 ng) were used as templates in 25 μl of qPCR reaction containing 12.5 μl TAKARA Mix PCR. The percentage of ssDNA (ssDNA%) generated by resection at selected sites was determined as previously described (22). Briefly, for each sample, a ΔCt was calculated by subtracting the Ct value of the not digested sample from the Ct value of the digested sample. The ssDNA% was calculated with the following equation: ssDNA% = 1/(2^ΔCt-1^ + 0.5) × 100.

### Chromatin immunoprecipitation experiments

Cells were crosslinked with formaldehyde (1%, 20 min) and ChIPs were performed as described (23) using 200 μg of chromatin. Briefly, nuclei were prepared and sonicated to obtain DNA fragments of ∼500–1000 bp. Following preclearing and blocking steps, samples were incubated overnight at 4°C with specific antibodies or without antibody as negative control. Immune complexes were then recovered by incubating the samples with blocked protein A/protein G beads for 2 h at 4°C on a rotating wheel. After extensive washing, crosslink was reversed by adding RNase A to the samples and incubating overnight at 65°C. After a 1h30 proteinase K treatment, DNA was purified with the GFX PCR kit (Amersham), and analyzed by Q-PCR.

### Real time PCR analysis

Q-PCR analysis was performed on a CFX96 Real-time system device (BioRad) using the IQ SYBR Supermix (BioRad Laboratories, Marnes-la-Coquette, France), according to the manufacturer's instructions. All samples were analyzed in triplicates.

### HPRT mutagenesis assay

Cells were transfected with the different siRNA then 48 h later irradiated. For determination of IR-induced mutagenesis, cells were irradiated (4 Gy) with a Cs^137^ source (Biobeam 8000), and let grow for 4 days. Then replica cultures were plated at the density of 10^6^ cells per plate and exposed to 20 μM of 6-thioguanine-containing media in order to determine the number of HPRT mutants. After 10 days, colonies were stained with crystal violet and counted. Mutation frequencies were calculated by correcting for plating efficiency and IR survival and expressed as mutation frequency per million of living cells.

### Clonogenic assay

Cells were transfected with the different siRNA and 48 h later plated at 500 cells/plate. Forty eight hours after siRNA transfection, cells were incubated with the PARP inhibitor (olaparib) at the indicated concentration and irradiated when mentioned. After 10 days, colonies were stained with crystal violet and counted. Colonies of more than 50 cells were scored.

### Karyotype analysis

Cells were transfected with the different siRNAs and 48 h later irradiated with 2 Gy when indicated. Twenty four hours after irradiation, cells were incubated in medium with colcemid (0.1 μg/ml) during 3 h. Cells were harvested, incubated in hypotonic buffer (75 mM KCl), and fixed with ethanol/acetic acid (3:1). Metaphase spreads were stained with DAPI. Chromosomal aberrations were analyzed using Leica microscope (DM5000) (objective 100×).

### Statistical analysis

Experimental differences were tested for significance using Student's *t*-test (two sidded) for two samples with paired samples. Unless indicated otherwise, results are the mean with error bars showing the standard deviation. In some cases (chromosomal aberrations analyses) significance for differences between two experimental conditions were tested using Wilcoxon test (two-sidded) and results are the mean with standard error.

## RESULTS

### NHEJ events are increased after p400 depletion

**Figure 1.**
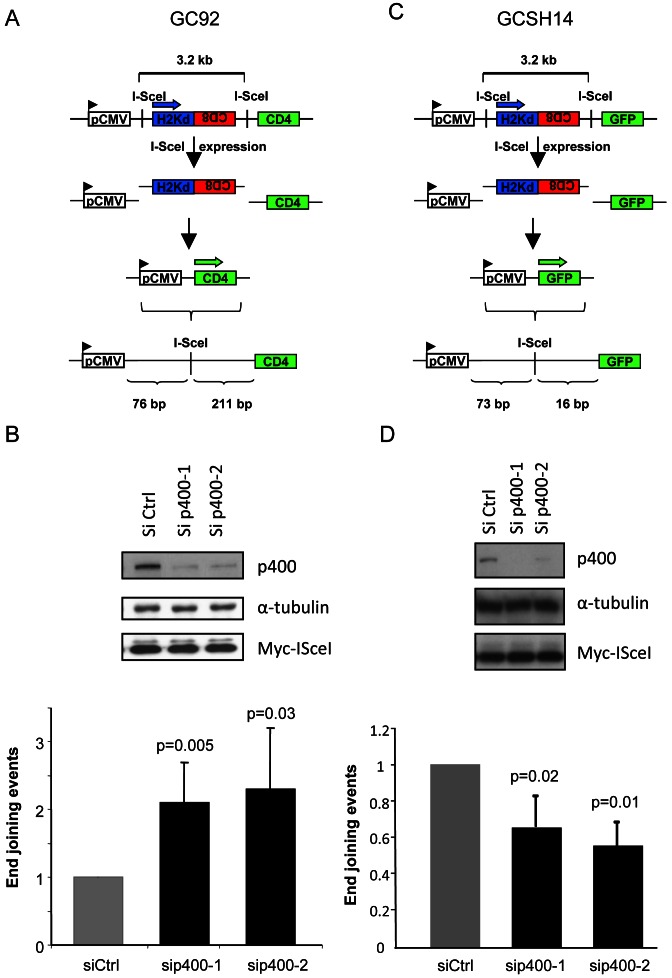
Effects of p400 depletion on NHEJ events. (**A**) Schematic representation of the NHEJ substrate present in GC92 cell line. (**B**) Frequency of NHEJ events in GC92 cells. For sip400-1, *n* = 5, for sip400-2, *n* = 4. Results are presented as the mean ± SD of independent experiments. (**C**) Schematic representation of the NHEJ substrate present in GCSH14 cell line. (**D**) Frequency of NHEJ events in GCSH14 cells. Results are the mean ± SD of five independent experiments.

To investigate the role of p400 in NHEJ, we used reporter systems for NHEJ relying on artificial substrates integrated in the genomic DNA, allowing the expression of a reporter gene following NHEJ-mediated repair of DSB induced by the I-SceI endonuclease. We first used the GC92 cell line which is derived from immortalized human fibroblasts and contains as a single copy a substrate composed of three genes H2-Kd, CD4 and CD8, the only expressed gene being H2-Kd (Figure 1A). Two I-SceI sites present in non-coding sequences are separated by 3.2 kb of DNA. After expression of and cleavage by the I-SceI enzyme, the H2-Kd/CD8 fragment is removed and the rejoining of DNA ends by NHEJ events leads to the expression of the CD4 gene (20,21). We observed that p400 depletion using two different siRNAs induces an increase in the frequency of CD4 positive cells monitoring NHEJ events, indicating that p400 represses NHEJ events (Figure 1B). Importantly, restoration of p400 expression using an expression vector coding for siRNA-resistant p400 restored normal level of NHEJ events (Supplemental Figure S1), demonstrating that the effects of p400 siRNA are specifically mediated by p400 depletion. Strikingly, when we used another cell line (GCSH14) with identical genetic background but harboring a different substrate designed to evaluate NHEJ activity (Figure 1C), we did not observe any increase of NHEJ events upon p400 depletion instead it induces a slight decrease in the efficiency of the repair (Figure 1D). The reason for this decrease is unclear, and probably related to changes in the local chromatin structure induced by p400 depletion.

Data from Figure 1 indicates that p400 specifically affects a step of end joining which is not observable with the latter substrate. The main difference between this latter substrate and the one present in GC92 cells is the distance between the repaired break and the important features for the expression of the reporter protein (the initiation codon for GFP) that is shorter in GCSH14 cell lines (Figure 1C) than in GC92 cells (Figure 1A). We reasoned that the differences observed in p400 dependency between these cell lines could result from large deletions around the breaks upon p400 depletion that would not be detected in GCSH14 cell lines as they would remove the GFP start codon located 16 bp from the I-SceI site. Such an explanation would also be consistent with our previous results showing that p400 depletion does not affect EJ events measured using another substrate harboring similar features than the GCSH14 cells (14).

### P400 depletion leads to large deletions around DSB

**Figure 2.**
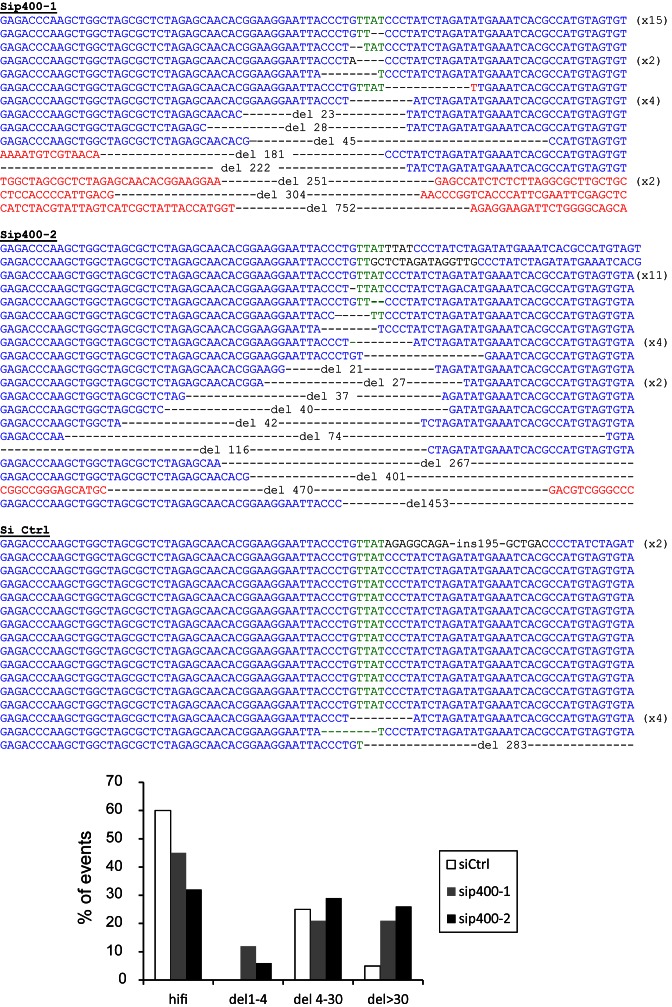
Sequences of the End-Joining repaired junctions. GC92 cells were transfected with Ctrl or p400 siRNAs then transfected with I-SceI to induce DSB. Seventy two hours after I-SceI transfection, genomic DNA was prepared and repaired junctions sequenced. Histogram showing the distribution of the repair events; HiFi: high fidelity, del 1–4 are deletions from the four protruding nucleotide generated by I-SceI cutting (green sequence). Sequences in black are insertions. Sequences in red are sequences outside the normal sequence in blue.

To confirm our assumption that the differences observed between the GC92 and GCSH14 result from large deletions around the repaired DNA breaks we collected the NHEJ induced junctions containing CD4 events generated in the GC92 cells, and sequenced them. We found that p400 depletion strongly increases the proportion of deletion events (Figure 2). Moreover, these deletions are larger than the ones observed in control cells (Figure 2 histogram). Such large deletions would not have been detected using the reporter substrate present in GCSH14 cell lines because the GFP start codon would have been deleted. Thus, the presence of larger deletions probably explains the difference observed after p400 depletion on NHEJ efficiency measured using the various reporter substrates. These data show that p400 prevents the occurrence of deletions at DSBs. They also show that the choice of the substrate to measure NHEJ activity can alter the meaning of the results obtained in such experiments based on reporter systems.

### P400 controls alternative EJ events and prevents genetic instability

**Figure 3.**
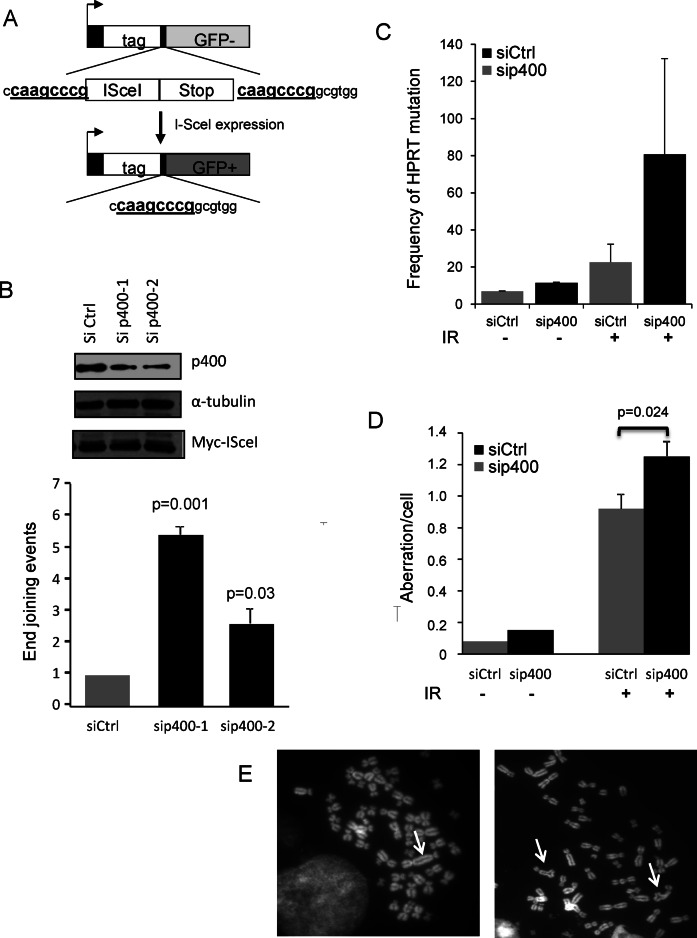
p400 depletion increases alternative End-Joining events and induces genetic instability. (**A**) Schematic representation of the alt-NHEJ substrate presents in U2OS cells. (**B**) Frequency of alt-NHEJ events in p400 depleted cells. Results are the mean ± SD of four independent experiments. (**C**) Mutagenesis at the HPRT locus in response to DNA damage in p400 depleted cells. HPRT mutagenesis in GC92 cells depleted for p400 compared with siCtrl treated cells. Transfected cells were left unirradiated or exposed to IR (4 Gy). Results are expressed as frequency of mutation per million living cells. Results are the mean and SEM of two totally independent experiments. (**D**) Transfected cells were unirradiated or irradiated (2 Gy) and 24 h later metaphase spreads prepared and chromosome aberrations per cell scored (*n* = 60). Results are expressed as mean ± standard error. Statistical analysis was performed using Wilcoxon test. (**E**) Examples of chromosomal aberrations observed on metaphase spreads, left panel arrow indicates dicentric chromosome, right panel arrows indicate triradial structures.

Next, we investigated the involvement of AltEJ pathway, which is a highly mutagenic pathway known to produce a high frequency of such deletion events (20,21). To examine the role of p400 in the control of AltEJ we used a cell line specifically designed to measure AltEJ events and derived from U2OS osteosarcoma cells (18). The substrate present in this cell line evaluates the use of microhomology (8 bp) to repair DSB as proposed for the AltEJ pathway (Figure 3A). Depletion of p400 with either of the two siRNAs increases the frequency of AltEJ events (Figure 3B), indicating that p400 represses AltEJ pathway and confirming that the occurrence of large deletions upon p400 inhibition probably results from the use of AltEJ pathways. We also tested the effect of p400 depletion on the use of single strand annealing (SSA), which is another backup repair mechanism. By using a U2OS cell line containing a substrate designed to monitor SSA events (18) we did not observe any increase in SSA efficiency upon p400 depletion, indicating that p400 does not repress the SSA process (Supplemental Figure S2).

As AltEJ is a highly mutagenic pathway, we measured the influence of p400 on genetic instability using the HPRT forward mutagenesis assay (24). Mutations or deletions that inactivatethe HPRT gene involved in the purine-salvage pathway, induce cell survival upon exposure to the purine analogue 6-thioguanine. The number of clones obtained in the presence of 6-thioguanine reflects the mutation rate on the endogenous HPRT gene. We observed that p400 depletion *per se* induces a 2-fold increase in the spontaneous mutagenesis frequency (Figure 3C). In response to IR exposure (4 Gy), mutagenesis frequency increases, as expected and is even more stimulated in p400-depleted cells. In addition, chromosomal aberrations such as dicentric chromosomes and chromatid breaks were scored and significantly increased in p400 depleted cells either untreated or after IR exposure (Figure 3D and E). Similar results were obtained in mouse embryonic fibroblasts from transgenic mice made defective for p400 (Supplemental Figure S3). These data indicate that p400 expression is important to maintain genetic stability both in basal conditions and in response to DNA damage, suggesting that the increased AltEJ activity in p400 depleted cells translates into genetic instability.

### P400 depletion leads to the recruitment of AltEJ factors to DSB

**Figure 4.**
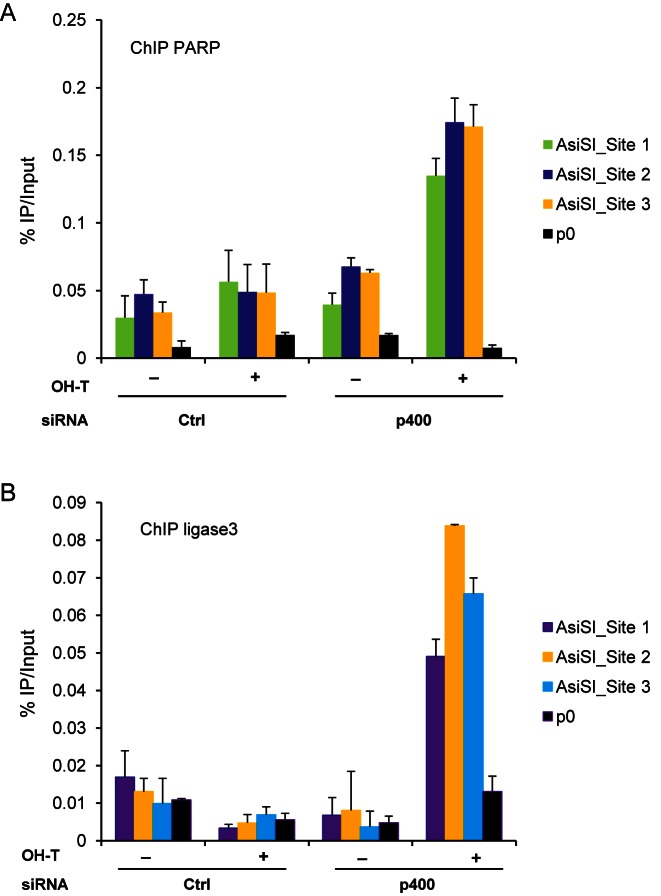
Influence of p400 on the recruitment of AltEJ factors at DSB. Chromatin immunoprecipitation (ChIP) experiment was performed on U2OS-AsiSI cells. DSB were induced by OHTam addition and ChIP experiment performed with PARP1 or DNA ligase3 antibodies. For each condition, three differents DSBs were examined together with the P0 as control. One representative experiment from three totally independent experiments is shown. Mean and standard deviation have been calculated on the PCR values. (**A**) Recruitment of PARP. (**B**) Recruitment of DNA ligase 3.

We next investigated consequences of p400 depletion on the recruitment of AltEJ factors to DNA double strand breaks. We previously established a cell line in which DSBs are induced by the relocalisation of the AsiSI endonuclease to the nucleus after addition of 4-hydroxy-tamoxifen (OHT) to the medium (19). Using this cell line we did not observe any specific enrichment of sequences near DSBs when PARP or DNA ligase3, two factors of AltEJ repair, (25–27) are immunoprecipitated from control cells (Figure 4A and B). However, in p400-depleted cells, immunoprecipitation of PARP and DNA ligase 3 leads to the co-immunoprecipitation of DNA sequences located close to three AsiSI-mediated DSBs. Such enrichment is not observed in absence of DSB (-OHT) nor for genomic sequences located far from any break (P0), indicating that it reflects binding of DNA ligase 3 and PARP to DSBs (Figure 4A and B). To our knowledge, these data are the first demonstration of recruitment of AltEJ factors close to DSB. In addition, they show that such recruitment can be detected only upon p400 depletion, demonstrating that p400 inhibits the recruitment of AltEJ factors to DSBs. Importantly, site 1 is classified as HR prone whereas site 2 has been shown as NHEJ prone by Aymard et al. (9). We confirmed the difference in the Rad51/XRCC4 ratio (Supplemental Figure S4) between these two sites. We found that the recruitment of AltEJ factors was comparable on these two sites upon p400 depletion, although it was slightly lower for the HR prone DSB (Figure 4A and B).

### AltEJ activation upon p400 depletion is due to the inhibition of late steps of homologous recombination

**Figure 5.**
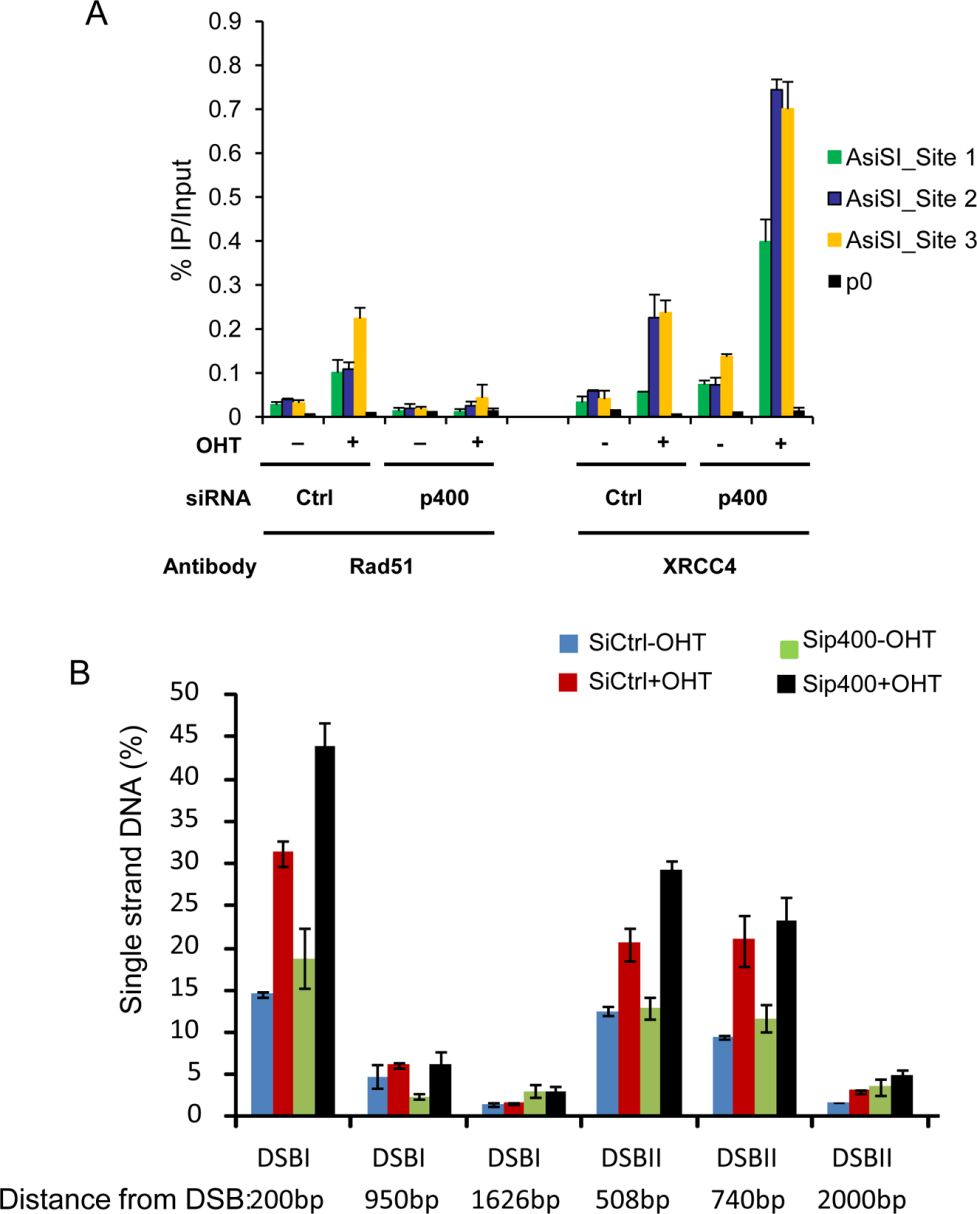
Influence of p400 depletion on the recruitment of HR and NHEJ factors at DSB. (**A**) Chromatin immunoprecipitation (ChIP) experiment performed on U2OS-AsiSI cells. DSBs were induced by OHTam addition and ChIP experiments were performed with Rad51 and XRCC4 antibodies. One representative experiment from three totally independent experiments is shown. For each conditions, three differents DSBs were examined together with the P0 as control. Mean and standard deviation have been calculated on the PCR values. (**B**) Generation of single strand DNA around DSB was evaluated in U2OS-AsiSI cells on two different DSBs. Cells were transfected with p400 siRNA and 48 h later DSBs were induced by OHT addition during 4 h. Typical experiment (from three independent experiment) is shown with mean and sd from PCR performed in triplicate.

We next investigated the mechanism by which p400 depletion leads to the appearance of AltEJ events. To gain insights into such mechanism, we first investigated the influence of p400 depletion on recruitment of HR and classical NHEJ factors. By Chromatin immunoprecipitation, we confirmed our previous findings that p400 depletion decreases the recruitment of Rad51 to DSB (Figure 5A). Strikingly, recruitment of a NHEJ factor, XRCC4 is not decreased upon p400 depletion. Actually, XRCC4 recruitment is increased, either because U2OS cells accumulate in G1 following p400 depletion (23), or because a subset of these breaks experience only minor resection and are redirected to classical NHEJ in the absence of p400. This result indicates that the increased recruitment of AltEJ factors correlates with a decreased recruitment of Rad51 upon p400 depletion, suggesting that some breaks normally repaired by HR are redirected to AltEJ in the absence of p400.

Interestingly, we previously showed that p400 depletion leads to defects in HR but does not alter RPA recruitment after DNA damage in U2OS and 293T cells, a finding that we confirmed here (Supplemental Figure S5) (14). We thus reasoned that the increased AltEJ activity upon p400 depletion could be the consequence of defective HR downstream of the DNA resection step. Indeed, such defects could result in the presence of large bunches of single strand DNA that cannot be repaired by classical NHEJ (which is impossible when resection is too important) nor by HR (since it is inhibited). To test this possibility, we first directly measured DNA resection efficiency upon p400 depletion. We used an assay recently developed for monitoring the presence of single strand DNA around DSB induced by sequence-specific endonuclease (22). We found that p400 depletion does not decrease the percentage of breaks harboring resection, neither the extent of resection (Figure 5B). Although it was not possible to examine DNA resection on the AsiS1 sites analyzed in ChIP experiment (because of unavailability of convenient restriction enzyme sites), these results indicate that the increase in AltEJ activity is not associated with a decrease in resection.

**Figure 6.**
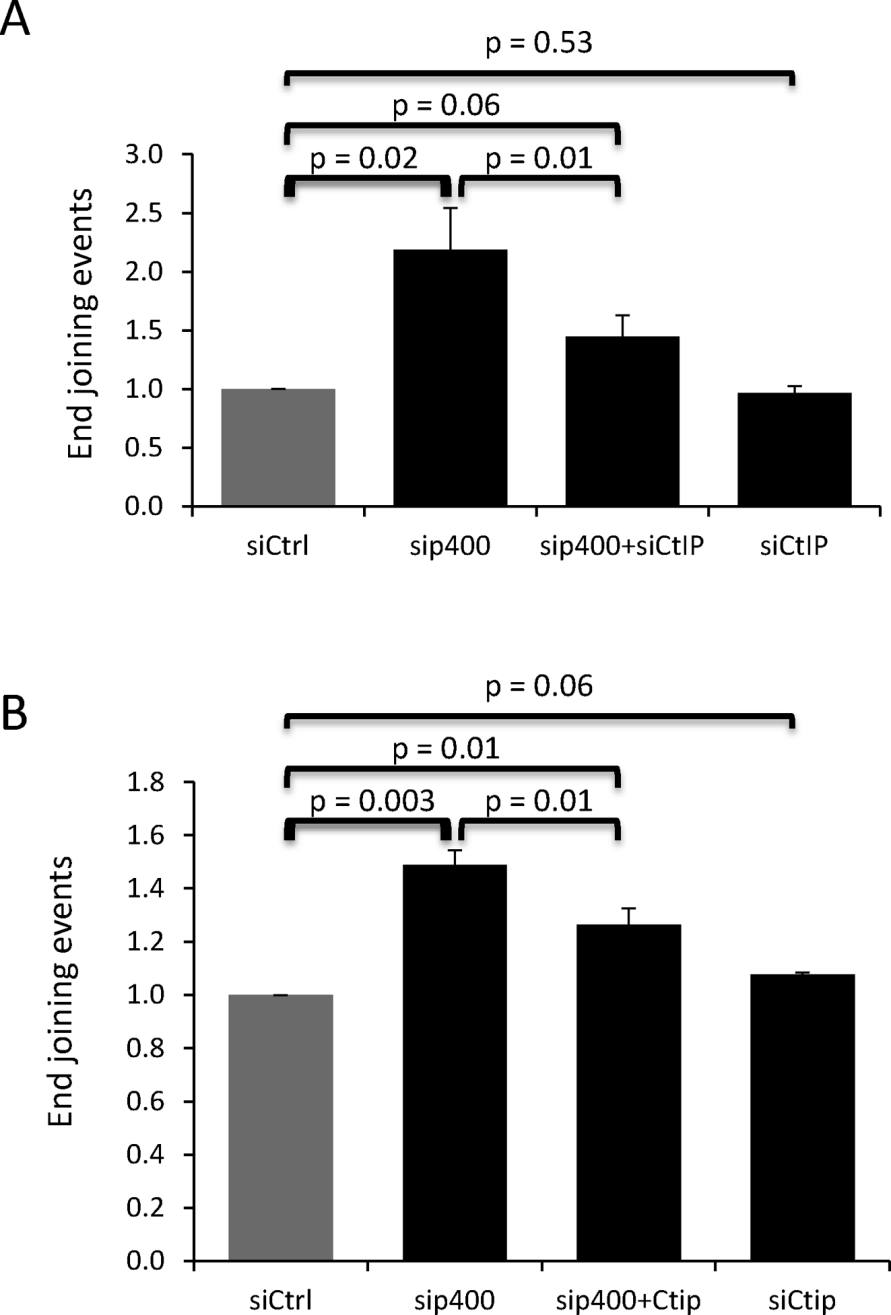
DNA resection via CtIP participates in the generation of p400 mediated-AltEJ events. (**A**) U2OS-EJ2 cells were transfected with p400 or CtIP siRNAs independently or co transfected with p400 and CtIP siRNAs then AltEJ events measured. Results are the mean ± SD of three independent experiments. (**B**) GC92 cells were transfected with p400 or CtIP siRNAs independently or co transfected with p400 and CtIP siRNAs then frequency of NHEJ events were measured. Results are the mean ± SD of three independent experiments.

We next tested the relationship between resection and AltEJ induction after p400 depletion. For that purpose, resection was inhibited using CtIP siRNAs (22), and we analyzed AltEJ events with the relevant reporter substrate (used in Figure 3A). We found that depleting p400, as already shown, stimulated AltEJ, whereas depleting CtIP has no effect, indicating that resection is not required for basal AltEJ (Figure 6A). However, codepletion of both largely abolishes the generation of AltEJ events caused by p400 depletion, indicating that the AltEJ events are dependent on CtiP-mediated resection around DSB. Similar results were obtained in GC92 cells, the cell line we used in Figure 1A allowing measurement of both NHEJ and AltEJ events showing that a significant part of AltEJ events induced following p400 deletion was decreased after co depletion of CtIP (Figure 6B). These data indicate that AltEJ events induced by p400 depletion are dependent on the presence of resected DNA ends.

### p400 defective cells are selectively killed by PARP inhibitor

**Figure 7.**
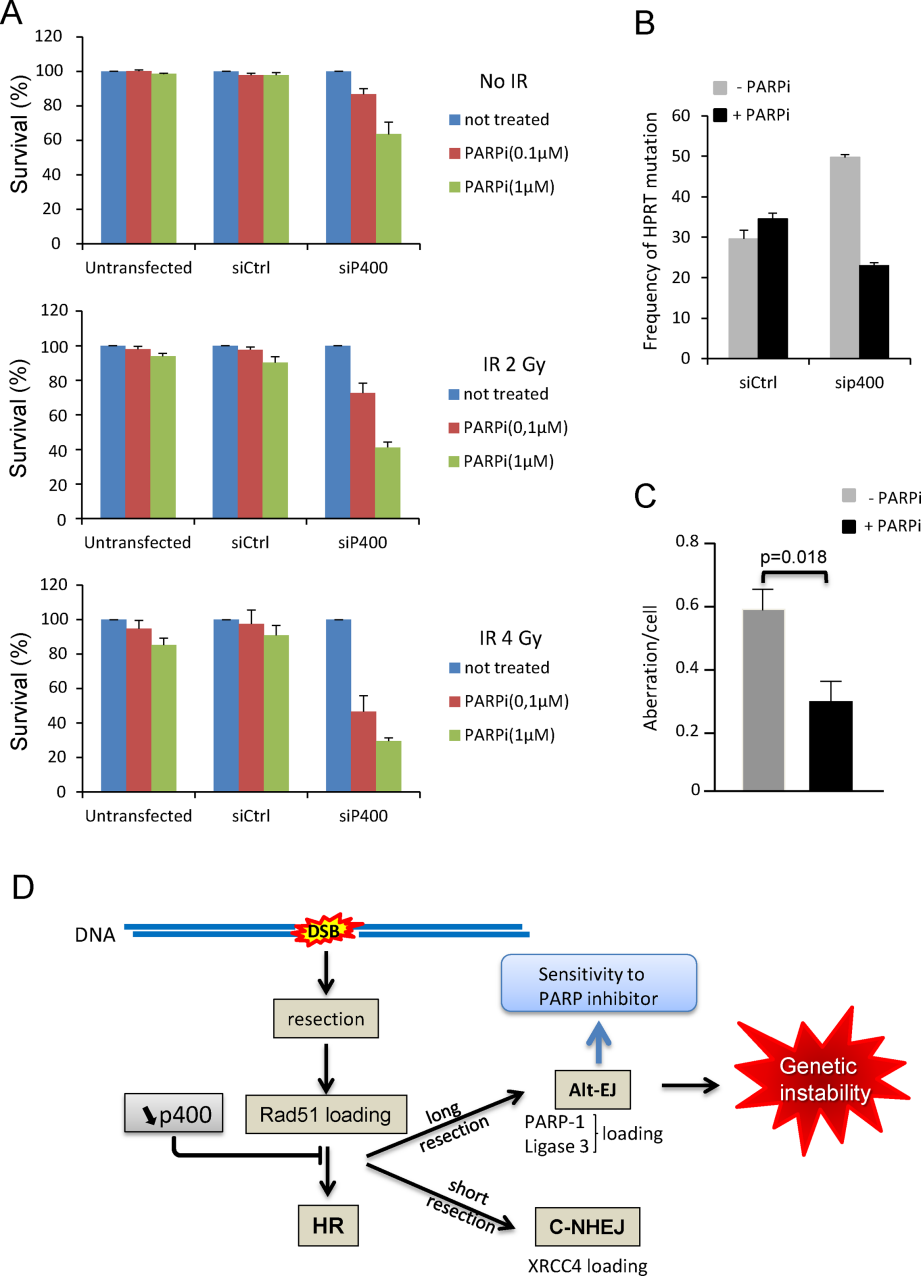
p400 deficient cells are selectively sensitive to PARP inhibition. (**A**) Cells transfected with Ctrl or p400 siRNAs were treated with the PARP inhibitor olaparib and exposed or not to ionizing radiations and let grow for 10 days before colonies counting. The number of colonies in untreated cells (without olaparib) was set at 1. Results are from one representative experiment performed in triplicate on which mean and sd were calculated. The same experiment was repeated in totally independent experiment. (**B**) Effect of PARP inhibition on HPRT mutagenesis. Cells transfected with Ctrl or p400 siRNAs were treated or not with olaparib (1 μM) and exposed to ionizing radiations then selected for HPRT mutants by 6-thioguanine treatment. Results are expressed as frequency of HPRT mutation per million living cells. Results are from one representative experiment performed in triplicate. The same experiment was repeated in totally independent experiment. (**C**) Effect of PARP inhibition on chromosomal aberrations. Cells transfected with p400 siRNAs were treated or not with olaparib (1 μM) and exposed to ionizing radiations (2 Gy). Metaphase spreads were prepared and aberrations scored. For sip400 transfected cells untreated and treated with PARP inhibitor (*n* = 52) and (*n* = 53) metaphases were examined, respectively. Results are presented as mean ± standard error. (**D**) schematic representation of the p400 role in controlling HR and Alt-EJ and its consequences on genetic instability.

Data shown above indicate that DSBs subjected to large resection are repaired by HR, or, if HR is defective, by AltEJ with the specific recruitment of PARP to DSBs. We reasoned that if the two pathways are made defective, these DSBs could be left unrepaired, leading to cell death. Such a mechanism could be the basis for the use of PARP inhibitors to kill selectively cancer cells as in the case of synthetic lethality observed for BRCA1 and BRCA2 deficiency (28,29) but also for deficiencies in other HR relevant factors such as rad51 (30). To test for a potential similar synthetic lethality, we treated p400 depleted cells with the PARP inhibitor olaparib (31) and performed a cell survival assay. We observed that p400 depleted cells exhibit sensitivity to PARP inhibition whereas at the same concentration there is no toxicity in untransfected cells and cells transfected with control siRNA (Figure 7A). To demonstrate that the synthetic lethality is dependent on DSBs, we treated cells with ionizing radiations. We found that the synthetic lethality between p400 depletion and PARP inhibition is much more prominent on irradiated cells (Figure 7A), indicating that the observed colethality is dependent on the presence of DSBs. Interestingly, PARP inhibitors are only effective on p400-depleted cells, suggesting that PARP inhibitors could be used to selectively eliminate cells harboring genetic instability resulting from p400 defect. Accordingly we show that treating p400-depleted cells with PARP inhibitor reverses IR-induced mutagenesis (Figure 7B) as well as genetic instability at the chromosomal level (Figure 7C) confirming their link with AltEJ. Moreover, it indicates that treatment with PARP inhibitor could be a very efficient way to get rid of cancer cells defective for p400.

## DISCUSSION

Previous works reported contradictory findings on the role of p400 on DSB repair by HR and NHEJ (14,15,32). These discrepancies could result from the approach used: siRNA vs shRNA, overexpression of mutant inactive protein but also from the reporter substrates used to measure DNA repair efficiency. Thus, we decided to use a panel of cell lines with different reporter systems in order to obtain a clear overview of the role of p400 in DSB repair. Using various cellular reporter systems designed for monitoring DSB repair by NHEJ, we obtained results which at first glance could be inconsistent. However, all these results could be reconciled after close examination of the specific features of each substrate together with sequencing experiments. Indeed, we discovered that p400 participates in the control of AltEJ pathway and prevents large deletions at DSBs to occur. In contrast to what was shown for H2A.Z, and in agreement with our previously published results, we did not find any effect of p400 depletion on classical NHEJ pathway.

Our data show that the use of several substrates should be recommended in future studies on DSB repair. In particular, we found that some substrates do not allow the detection of large deletion events, since such events result in the removal of features required for the expression of the reporter gene (promoters or ATG, for example). As a consequence, the use of these reporter substrates underestimates NHEJ-dependent events, and may prevent the observation of pathways leading to large deletions, such as the AltEJ pathway. Reassessment of previous results obtained using such reporter systems could be useful. We further show the recruitment of AltEJ factors (PARP and DNA ligase 3) to specific DSB (in the context of p400 depletion, see Figure 4). To our knowledge, this is the first demonstration of such recruitment. Our data could thus represent the bases for future studies dedicated to investigate the determinants of the recruitment of AltEJ factors to DSB. Altogether, these data indicate that p400 could be of particular importance for controlling DSB repair and could be seen as a brake to AltEJ use. We also provide important insights into the mechanisms by which p400 prevents the use of AltEJ: First the increase of AltEJ is blocked by the inhibition of resection. Moreover, recruitment of AltEJ proteins correlates with the decrease in the recruitment of Rad51, a key factor of the HR pathway. We also previously showed that formation of BRCA1 foci, another feature of HR is defective upon p400 depletion (14). These data indicate that AltEJ functions in p400 depleted cells as a back-up pathway to repair DSBs that are normally repaired by HR. We therefore propose the following model for the role of p400 in DSB repair (Figure 7D). In the absence of p400, resection takes place normally on the DSB that should be repaired by HR (see model on Figure 7D). HR-mediated repair is not completed due to the lack of loading of Rad51 on single strand DNA. Although some breaks with minor resection are probably redirected towards classical NHEJ the back-up pathway AltEJ takes charge of DSB harboring large resection, leading to large deletions (21,33). It should be noted that we do not observe Pol Q dependent insertions, that were recently shown to occur when HR is not functional and replaced by AltEJ showing genetic interaction between HR and AltEJ through pol Q (34,35). It suggests that the AltEJ mechanism observed upon p400 depletion is different from the mechanisms observed upon depletion of factors of the HR pathway. The reason for this difference is unclear for the moment, but could be related on the effects of p400 on specific gene transcription, which may result in changes in the expression of some AltEJ factors or regulators, leading to a slightly different mutagenic signature with different processing of the breaks depending on the context. We can speculate that the increase in the recruitment of AltEJ factors is not only due to the presence of single strand DNA but indeed p400 actively block PARP and DNA ligase3 loading onto chromatin. This assumption is supported by the observation that rad51 depletion (decreasing HR but not perturbing resection and ssDNA generation) did not lead to PARP1 and DNA ligase3 recruitment to DSB (Supplemental Figure S8). To check the potential involvement of G1 arrest and the channelling to AltEJ because G1 cells are not loading Rad51, we evaluated the cell cycle profile of U2OS EJ2 cells after p400 depletion and observed few effect on cell cycle arrest in G1 (Supplemental Figure S9). These results were reinforced by performing co depletion of p21 and p400 to abrogate potential G1 block showing that after p21 depletion there still increased level of AltEJ events. Together these results show that potential block in G1 influenced only marginally the AltEJ events induced after p400 depletion. Our data demonstrate that AltEJ inhibition by p400 is not totally due to defects in the HR pathway or change in cell cycle distribution but rather represents the ability of p400 to interfere or control AltEJ processes.

Strikingly, we do not find any effect of Tip60 depletion on AltEJ activity measured using the two different cell systems present in U2OS EJ2 and GC92 cells (Supplemental Figure S6). However, a recent study found that Tip60 depletion decreased the occurrence of AltEJ events (36). These data could indicate that the role of p400 in the AltEJ process is independent of the Tip60 complex. Alternatively, the function of Tip60 in DNA damage signaling through acetylation of ATM (37) could prevent any analysis of its role in later events of DNA damage repair.

Importantly, our results could explain, at least in part, the concept of synthetic lethality between PARP inhibitors and defective HR (through mutations in BRCA1) as well as the relative absence of clear link between PARP inhibition, base excision repair (BER) and toxicity. Synthetic lethality with PARP inhibitor has been attributed to the combined defect in HR pathway together with the inhibition of the repair of endogenous DNA damage by PARP and BER generating single strand breaks. However, the absence of effects of XRCC1 depletion (an actor of the BER pathway acting at the first step of the process) raises the question of the BER activity via PARP in the efficiency of PARP inhibitor to kill cells (38). One possible explanation was given by Patel et al. describing the potential involvement of NHEJ in this process (39). However, they reported that NHEJ defects diminish the PARP inhibitor lethality. These results can be reconciled in view of our results. DNA breaks that should be repaired by HR are redirected to AltEJ when HR is defective (in BRCA1 mutants for example), in a mechanism involving PARP. The use of PARP inhibitors would inactivate this backup pathway, and DSBs would remain unrepaired, leading to cell death. As a consequence, the efficiency of PARP inhibitors would be mediated by the role of PARP in AltEJ and its recruitment to DSB rather than its general role on BER.

We previously showed that colon cancers show defects in p400 expression (40). Such observation could highlight a new target to treatment using PARP inhibitors because of defective HR and because of the increased need of PARP to repair DSB by AltEJ. More generally, our data suggest that p400 deficient cells could be efficiently suppressed by PARP inhibitors. As such, the p400 status of cancer cells could have predictive clinical value to orientate therapy towards PARP inhibitors.

## Supplementary Material

SUPPLEMENTARY DATA
