# Supplementary material for: Control of alternative end joining by the chromatin remodeler p400 ATPase
Source: Nucleic Acids Res. 2015 Nov 17;44(4):1657–68. doi: 10.1093/nar/gkv1202 (PMC4770216; doi:10.1093/nar/gkv1202)
Supplement: SUPPLEMENTARY DATA [file supp_44_4_1657__index.html]

Control of alternative end joining by the chromatin remodeler p400 ATPase — SUPPLEMENTARY DATA 

# Control of alternative end joining by the chromatin remodeler p400 ATPase

## SUPPLEMENTARY DATA

- SUPPLEMENTARY DATA
