# Supplementary material for: Control of alternative end joining by the chromatin remodeler p400 ATPase
Source: Nucleic Acids Res. 2015 Nov 17;44(4):1657–68. doi: 10.1093/nar/gkv1202 (PMC4770216; doi:10.1093/nar/gkv1202)
Supplement: SUPPLEMENTARY DATA [file supp_gkv1202_nar-02723-d-2015-File009.pdf]

A

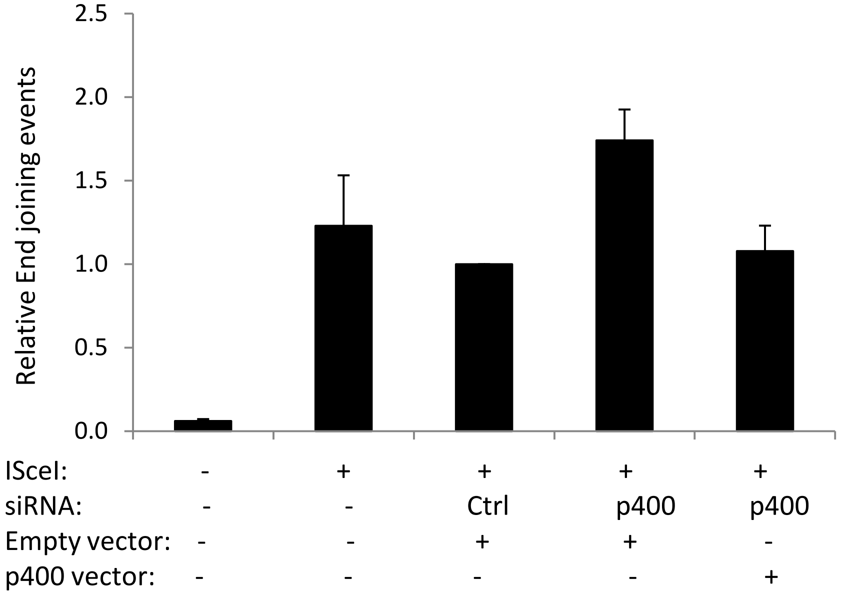

B

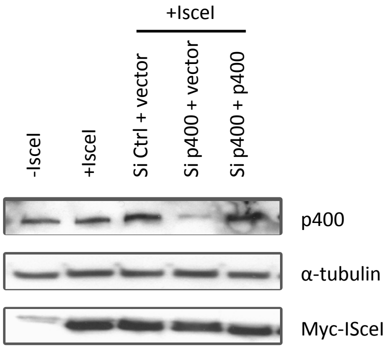

Supplemental Figure S1: Complementation experiment in GC92 cells using siRNA against p400 and p400 plasmid unsensitive to p400 siRNA. (A) relative NHEJ activity in GC92 cells. (B) Western blot showing the efficiency of p400 depletion and its complementation by expression of a p400 coding plasmid. The level of I-SceI expression is also shown. Results are the mean +/- sd from three independent experiments

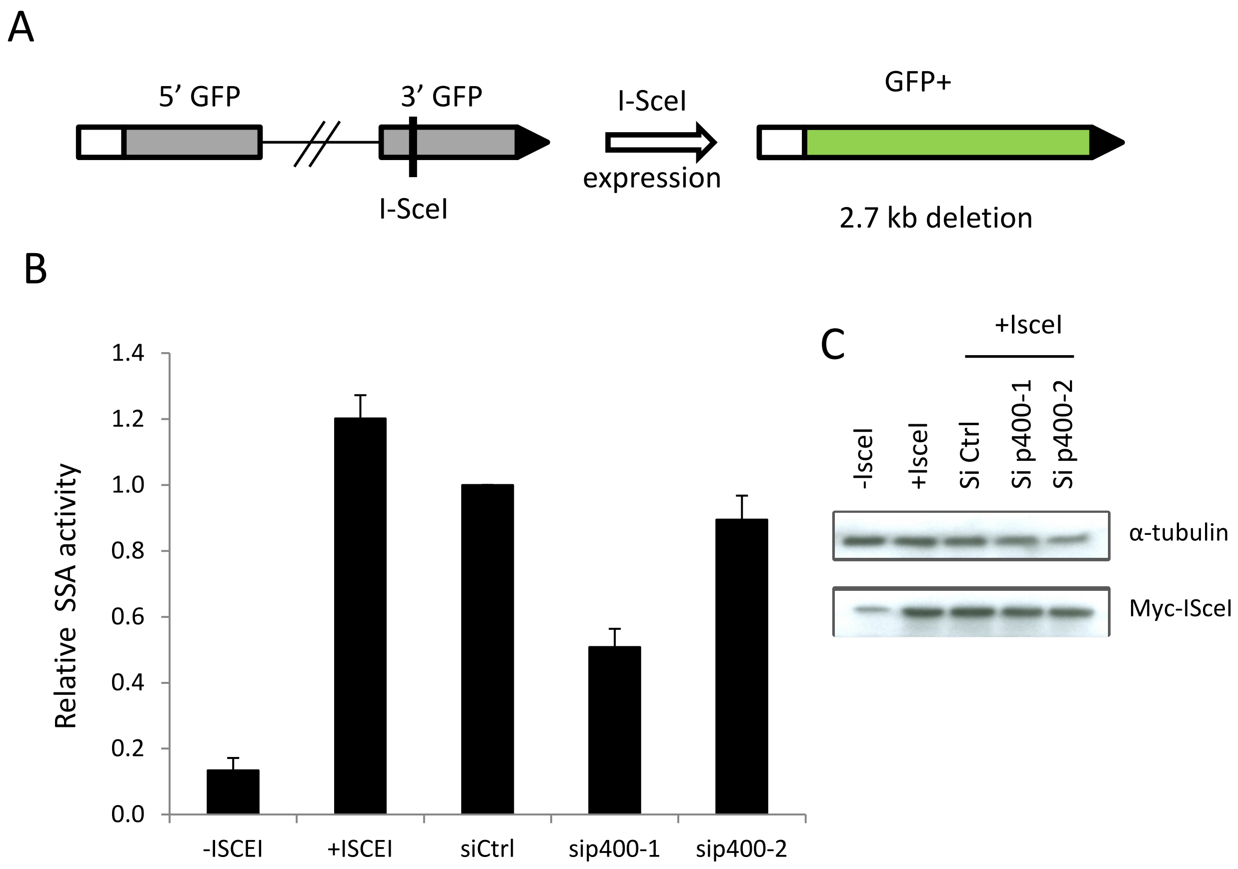

Supplemental Figure S2: Effect of p400 depletion on single strand annealing (SSA) activity using an U2OS cell line containing specific substrate designed to measure SSA.
(A) Schematic representation of the substrate designed to measure SSA stably integrated in cells (Bennardo et al 2008) (B) measurement of SSA activity. Results are the mean +/- sd from 3 independent experiments. (C) Western blot showing I-SceI expression.

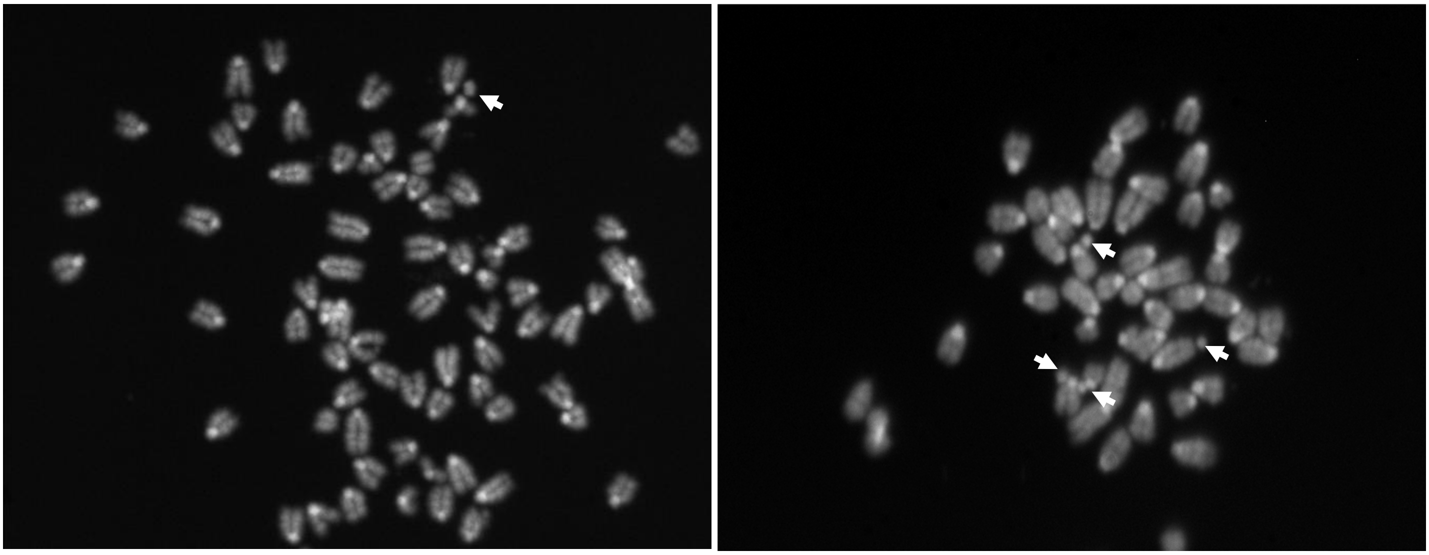

p400 $^{+/+}$ p400 $^{\Delta/\Delta}$

	**unirradited**	**irradiated**
p400 $^{+/+}$	10/761 (1.5%)	44/752 (5.8%)
p400 $^{\Delta/\Delta}$	25/440 (5.7%)	24/227 (10.6%)

Supplemental Figure S3: Genetic instability in mouse embryonic fibroblasts from transgenic mice defective for p400 (Ueda et al 2007). Upper panel: examples of metaphase spreads from p400 $^{+/+}$ and p400 $^{\Delta/\Delta}$ MEFs (arrows indicate pieces of broken chromosome). Lower panel: quantification of the number of chromosomes breaks. Percentages are expressed as the ratio of chromosomes breaks vs the total number of chromosomes examined. Counting were performed on unirradiated cells and after ionizing radiations exposure (4Gy).

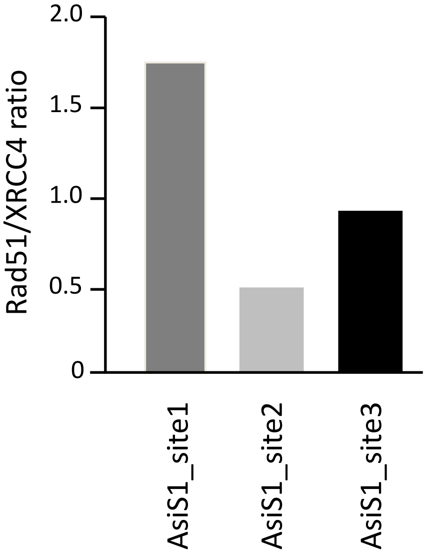

Supplemental Figure S4: Classification of the AsiS1 site according to the ratio of rad51 vs XRCC4 binding after DSB induction. Results are from ChiP experiment performed on U2OS cells after DSB induction by OHTam treatment. Graph is generated from results obtained in ChIP experiments in Figure 4. From these experiments site 1 is considered as a HR prone DSB, site 2 as a NHEJ prone and site 3 as a non preferential site.

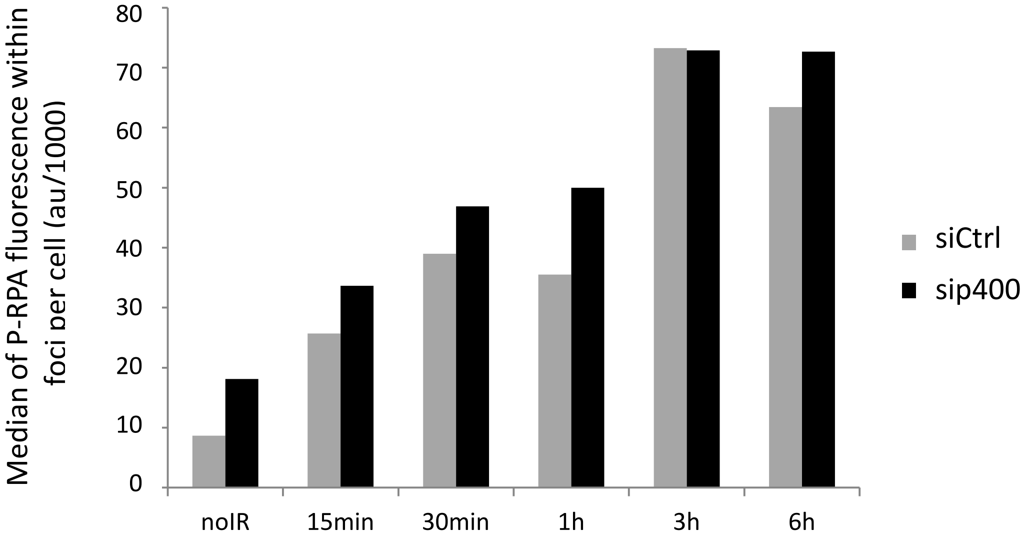

Supplemental Figure S5. Analysis of phospho-RPA generation in U2OS cells in response to IR (8Gy) in p400 depleted cells. Analysis was performed by automated fluorescence analysis using high content Screening system (Operetta, Perkin Elmer). Analysis was performed on at least 200 nuclei for each conditions.

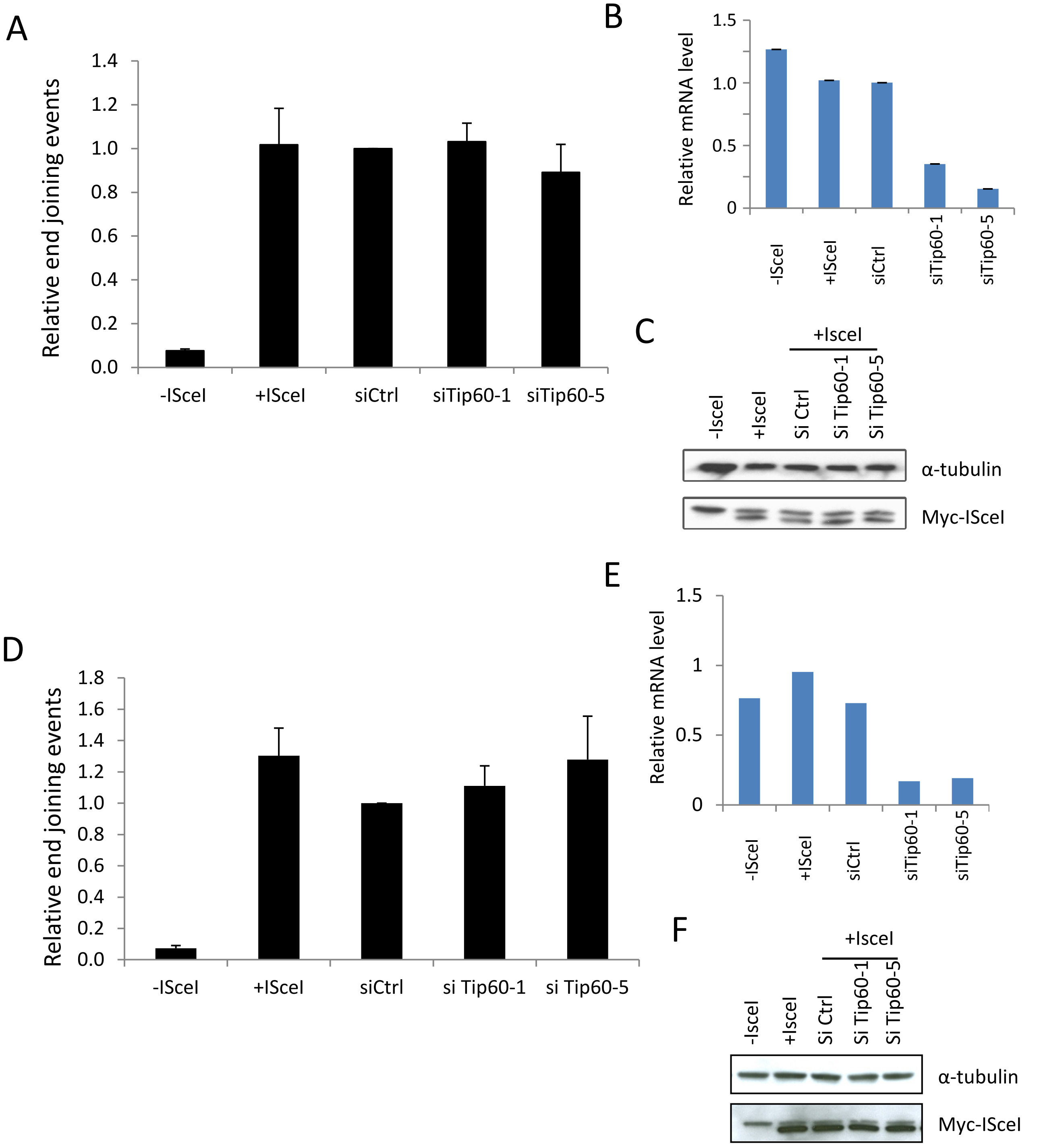

Supplemental Figure S6: Effect of Tip60 depletion on NHEJ activity.
(A) Relative end joining activity in GC92 cells. Results are the mean +/- sd from 3 independent experiments (B and E) Efficiency of Tip60 depletion evaluated at the mRNA level by RT-qPCR. (C and F) Western blot showing I-SceI expression in the different conditions. (D) relative end joining activity in U2OS EJ2 cells . Results are the mean +/- sd from 3independent experiments

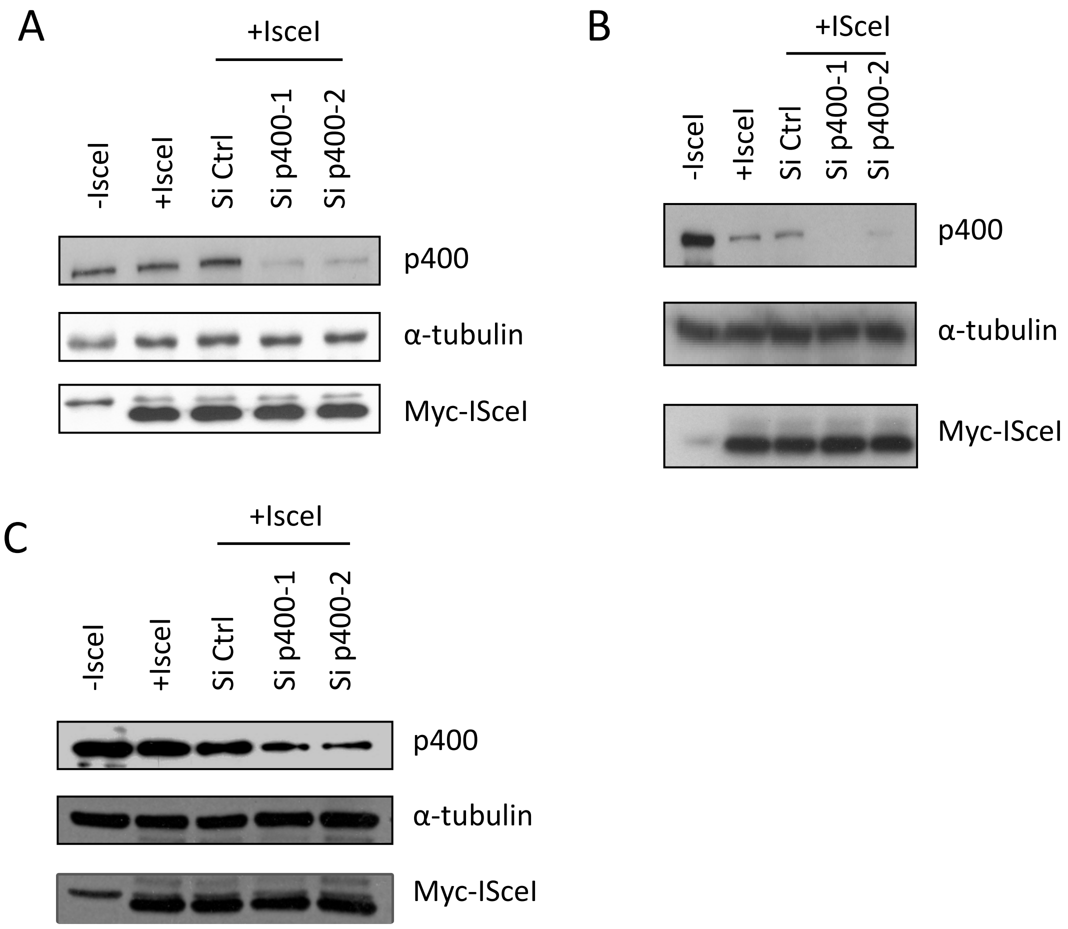

Supplemental Figure S7. Western blot showing the specificity of the I-SceI expression through detection of the myc tag . (A) Western blot relative to figure 1B in GC92 cells. (B) Western blot relative to figure 1D in GCSH14 cells (C) Western blot relative to figure 3B in U2OS cells.

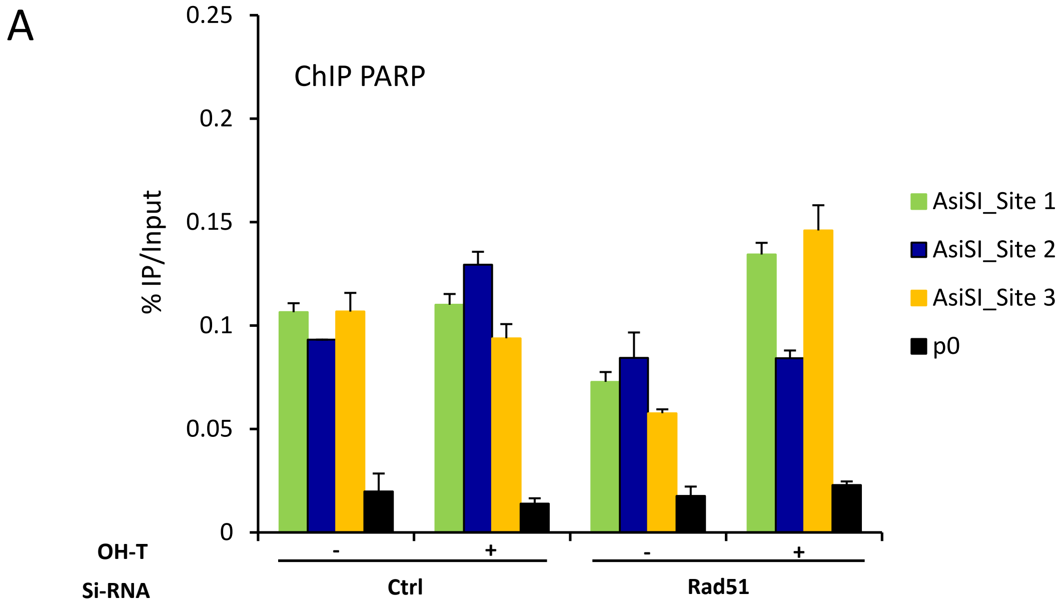

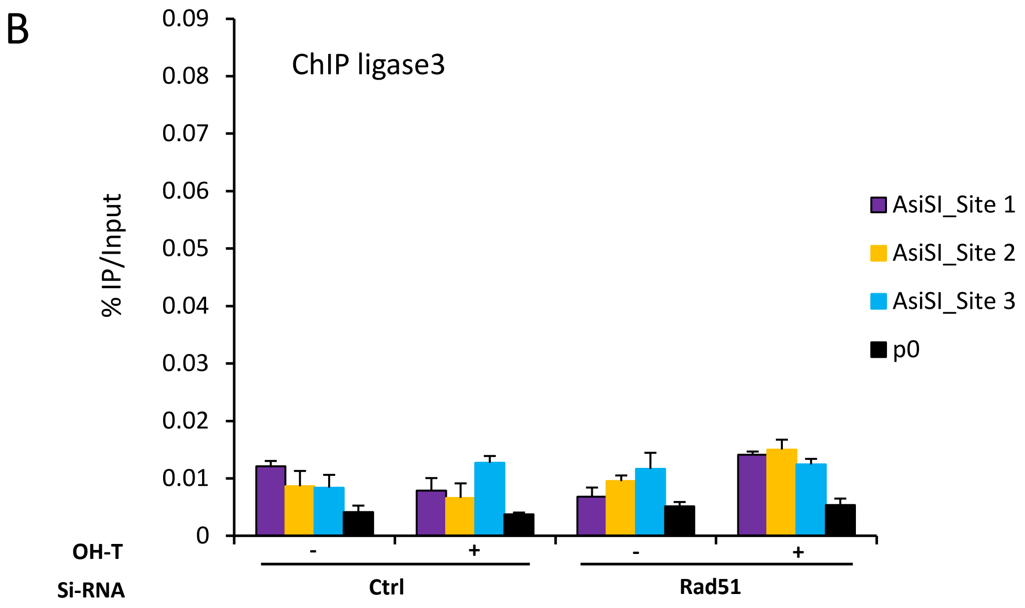

Supplemental Figure S8. Chromatin immunoprecipitation after rad51 siRNA mediated depletion. Chromatin immunoprecipitation (ChIP) experiment was performed on U2OS-AsiSI cells transfected with the indicated siRNA (previously validated in Courilleau et al.). DSB were induced by OHTam addition and ChIP experiment performed with PARP1 or DNA ligase3 antibodies. For each condition 3 differents DSBs were examined together with the P0 as control. One representative experiment from 2 totally independent experiments is shown. Mean and standard deviation have been calculated on the PCR values. (A) Recruitment of PARP. (B) Recruitment of DNA ligase 3. Note that no specific signal in PARP1 and DNA ligase3 ChIP is observed in Rad51 depleted cells upon DSB induction (+ OH-T).

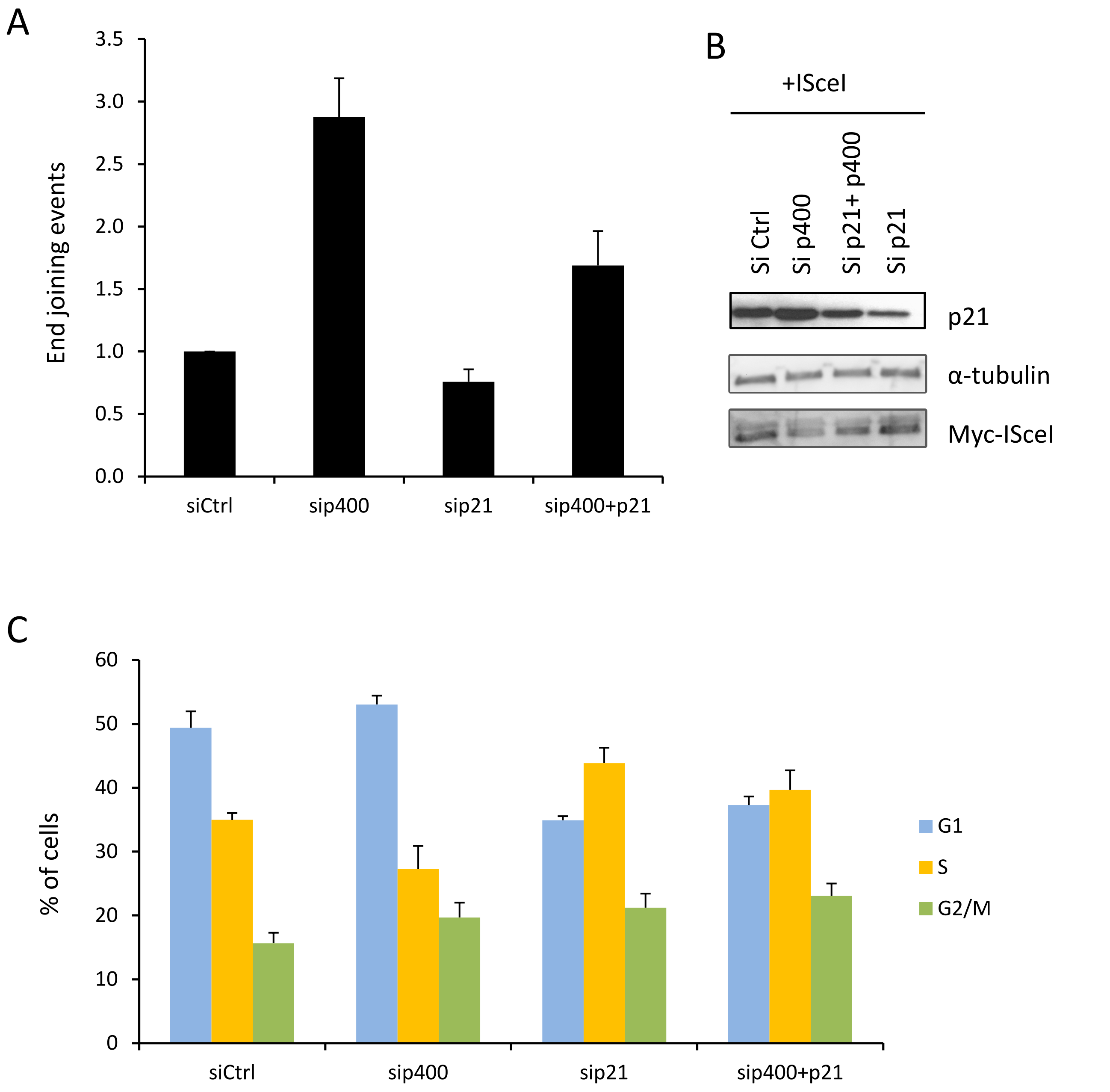

Supplemental Figure S9. (A) Effect of p21 depletion on the frequency of alt-NHEJ events in p400 depleted cells. U2OS cells bearing AltEJ substrate (U2OS EJ2) were transfected with p21 or p400 siRNAs or with a combination of both siRNAs. 48h later cells were transfected with I-SceI to induce DSB. Results are the mean +/- sd of 3 independent experiments. (B) Western blot showing p21 and I-SceI expression. Note that p21 expression increased after p400 depletion as expected (Tyteca et al 2006, fig8) showing the efficiency of p400 siRNA. (C) Cell cycle distribution. Results are the mean+/- sd of 3 independent experiments.

Supplemental Materials and Methods

Analysis of Phosphorylated RPA

Cells were seeded in 96 well plates at a density of xxx and transfected with the indicated siRNA Using INTERFERIN according tp the manufacturer's instructions (Ozyme). Forty eight hours after Transfection cells were irradiated with ionizing radiations (8 Gy) and fixed with PFA at different time post IR.

SSA measurement

U2OS SSA cells were transfected with siRNA (10 nM) using Interferin (Ozyme) according to the manufacturer's instructions. 24 hours after, cells were transfected with I-SceI coding plasmid using JetPei (Ozyme) according to the manufacturer's instructions. 72 hours after, cells were trypsinized and analyzed by flow cytometry (BD Facscalibur) to detect GFP expressing cells. Percentage of GFP positive cells was calculated after analysis on 25000 sorted events.

Effect of p21 depletion on AltEJ events

U2OS EJ2 cells were transfected with the same amount of siRNA (20 nM) in each conditions. 10 nM of siCtrl was added in p21 and p400 to keep the final concentration of siRNA identical in all samples. The following of the procedure was identical to the one described in materials and methods.

Sequences of the siRNAs used

UGAAGAAGGUUCCCAAGAA-dTdT : sip400-1
CGACACAUUGGAUACAGAA-dTdT : sip400-2
ACGGAAGGUGGAGGUGGUU-dTdT : siTip60-1
GGACGUAAGAACAAGAGUU-dTdT : siTip60-5
CGUACGCGGAAUACUUCGA-dTdT : siCtrl
GCUAAAACAGGAACGAAUC-dTdT : siCtiP

Supplemental references

Bennardo, N., Cheng, A., Huang, N., and Stark, J.M. (2008). Alternative-NHEJ is a mechanistically distinct pathway of mammalian chromosome break repair. PLoS Genet *4*, e1000110.

Ueda, T., Watanabe-Fukunaga, R., Ogawa, H., Fukuyama, H., Higashi, Y., Nagata, S. , and Fukunaga, R. (2007). Critical role of the p400/mDomino chromatin-remodeling ATPase in embryonic hematopoiesis. Genes Cells *12*, 581-592.
